# Enhanced Anti-Atherosclerotic Efficacy of pH-Responsively Releasable Ganglioside GM3 Delivered by Reconstituted High-Density Lipoprotein

**DOI:** 10.3390/ijms222413624

**Published:** 2021-12-20

**Authors:** Tong Rong, Bo Wei, Meiying Ao, Haonan Zhao, Yuanfang Li, Yang Zhang, Ying Qin, Jinhua Zhou, Fenfen Zhou, Yong Chen

**Affiliations:** 1 College of Life Sciences, Nanchang University, 999 Xuefu Ave, Honggutan District, Nanchang 330031, China; rong_tong@139.com (T.R.); weibo940222@foxmail.com (B.W.); qinying1013@hotmail.com (Y.Q.); Zff15079134005@163.com (F.Z.); 2 Jiangxi Key Laboratory for Microscale Interdisciplinary Study, Institute for Advanced Study, Nanchang University, Nanchang 330031, China; 15291855060@163.com (H.Z.); liyuanfang0504@163.com (Y.L.); yang_161129@163.com (Y.Z.); 15797760311@163.com (J.Z.); 3 School of Basic Medical Sciences, Jiangxi University of Chinese Medicine, Nanchang 330025, China; 15870687101@163.com

**Keywords:** ganglioside GM3, atherosclerosis, reconstituted high-density lipoprotein (rHDL), drug delivery system, ApoA-I, SR-B1

## Abstract

Recently, the atheroprotective role of endogenous GM3 and an atherogenesis-inhibiting effect of exogenous GM3 suggested a possibility of exogenous GM3 being recruited as an anti-atherosclerotic drug. This study seeks to endow exogenous GM3 with atherosclerotic targetability via reconstituted high-density lipoprotein (rHDL), an atherosclerotic targeting drug nanocarrier. Unloaded rHDL, rHDL loaded with exogenous GM3 at a low concentration (GM3_L_-rHDL), and rHDL carrying GM3 at a relatively high concentration (GM3_H_-rHDL) were prepared and characterized. The inhibitory effect of GM3-rHDL on lipid deposition in macrophages was confirmed, and GM3-rHDL did not affect the survival of red blood cells. In vivo experiments using ApoE^−/−^ mice fed a high fat diet further confirmed the anti-atherosclerotic efficacy of exogenous GM3 and demonstrated that GM3 packed in HDL nanoparticles (GM3-rHDL) has an enhanced anti-atherosclerotic efficacy and a reduced effective dose of GM3. Then, the macrophage- and atherosclerotic plaque-targeting abilities of GM3-rHD, most likely via the interaction of ApoA-I on GM3-rHDL with its receptors (e.g., SR-B1) on cells, were certified via a microsphere-based method and an aortic fragment-based method, respectively. Moreover, we found that solution acidification enhanced GM3 release from GM3-rHDL nanoparticles, implying the pH-responsive GM3 release when GM3-rHDL enters the acidic atherosclerotic plaques from the neutral blood. The rHDL-mediated atherosclerotic targetability and pH-responsive GM3 release of GM3-rHDL enhanced the anti-atherosclerotic efficacy of exogenous GM3. The development of the GM3-rHDL nanoparticle may help with the application of exogenous GM3 as a clinical drug. Moreover, the data imply that the GM3-rHDL nanoparticle has the potential of being recruited as a drug nanocarrier with atherosclerotic targetability and enhanced anti-atherosclerotic efficacy.

## 1. Background

Atherosclerosis is a lifelong cardiovascular disease which is the leading cause of death and disability globally by triggering stroke, coronary artery disease, and other artery diseases. Occurrence of atherosclerotic lesions/plaques in artery walls is one of the most fundamental features of atherogenesis. Ganglioside GM3 is the simplest member of the sialic acid-bearing glycosphingolipid family which is mainly present on cell surfaces and potentially plays important roles in multiple cellular activities (e.g., the recognition, binding, adhesion, and migration of cells), signaling pathways, and diseases (e.g., cancers and obesity) [1,2,3,4]. Gangliosides are also present in all vascular cell types in various amounts/compositions, and their role in vascular pathogenic mechanisms is still debated [5,6,7]. The significant increases of GM3 concentration both in the extracellular matrix and in the plasma membrane of cells in atherosclerotic plaques have been reported previously [8,9,10,11] as well as the significantly elevated expression of GM3 synthase in atherosclerotic plaques [6,12]. This evidence implies the potential correlation of ganglioside GM3 with atherosclerosis. 

Recently, we revealed that exogeneous GM3 can increase the size, zeta potential, and charge of low-density lipoprotein (LDL) particles, impair LDL oxidation, monocyte-endothelial cell adhesion, LDL recognition/internalization by cells, and lipid deposition in macrophages, and inhibit the development of atherosclerotic plaques in ApoE^−/−^ mice fed a high fat diet [13]. We also found that the downregulation of endogenous GM3 via RNA interference of GM3 synthase expression enhances the monocyte-endothelial cell adhesion [13]. These data suggest an atheroprotective role of endogenous GM3 (it probably is a self-protection mechanism of vessel walls against atherogenesis) and an inhibitory effect of exogenous GM3 on atherogenesis, raising the possibility of finding a new anti-atherosclerotic drug [13]. However, exogenous GM3 will enter the atherosclerotic plaques in limited amount only. Endowing exogenous GM3 with atherosclerotic targetability is an important work for the development/application of GM3 as a clinical drug. 

Reconstituted high-density lipoprotein (rHDL) is a synthetic form of endogenous HDL which is composed of lipids (mainly free/esterified cholesterol and phospholipids) and apolipoproteins (ApoA-I is the major apolipoprotein) as one of the five human plasma lipoproteins and functions as a natural cholesterol delivering nanocarrier to remove excess cholesterol from peripheral tissues/cells (this process generally is named as reverse cholesterol transport or RCT) [14,15]. In recent years, due to multiple advantages (e.g., biocompatibility, nonimmunogenicity, long circulation time, specific recognition by receptors, among others), an rHDL nanoparticle has been developed as a therapeutic delivery system for different drugs [16,17,18,19,20], particularly for the anti-atherosclerotic drugs due to the atherosclerotic plaque-targeting ability of rHDL [21,22,23,24,25].

In this study, a drug delivery system rHDL nanoparticle was applied to transport ganglioside GM3 and target atherosclerotic lesions for improving the anti-atherosclerotic efficacy of exogenous GM3. This lowered the efficient dose of GM3 in ingredients, and impaired its potential side effects. Moreover, we found that the acidic environment in atherosclerotic plaques may help with the release of GM3 from the rHDL nanoparticle.

## 2. Results

### 2.1. Characterization of GM3-rHDL Nanoparticles

**Figure 1:**
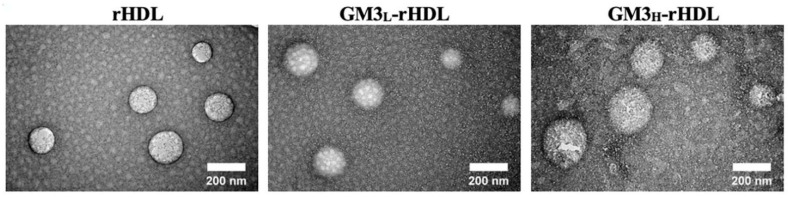
TEM images of rHDL, GM3_L_-rHDL, and GM3_H_-rHDL nanoparticles, respectively.

The rHDL nanoparticles loaded with or without exogenous GM3 at a low or high concentration (i.e., rHDL, GM3_L_-rHDL, and GM3_H_-rHDL nanoparticles, respectively) were prepared via the thin film dispersion method. A dynamic light scattering analyzer detected that the average size/diameter of rHDL nanoparticles is ~161 nm, while the GM3-loaded rHDL nanoparticles have a slightly increasing average diameter (~177 nm and ~210 nm for GM3_L_-rHDL and GM3_H_-rHDL, respectively) (Table 1) which was further confirmed by the transmission electron microscopic images (Figure 1). The GM3 entrapment efficiency (EE) of both GM3_L_-rHDL and GM3_H_-rHDL was >80% (Table 1). The drug loading efficiency (DL) of GM3_L_-rHDL was relatively low (<1%) whereas GM3_H_-rHDL had an average GM3 loading efficiency of ~3.4% (Table 1). 

### 2.2. Confirmation of the Inclusions of ApoA-1 and GM3 in Nanoparticles

**Figure 2:**
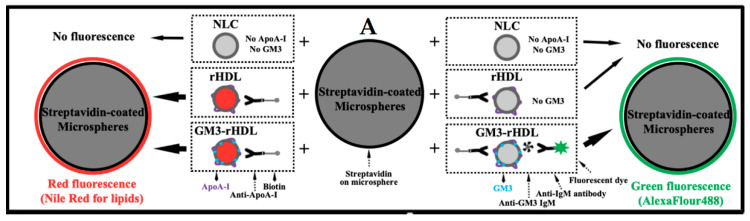

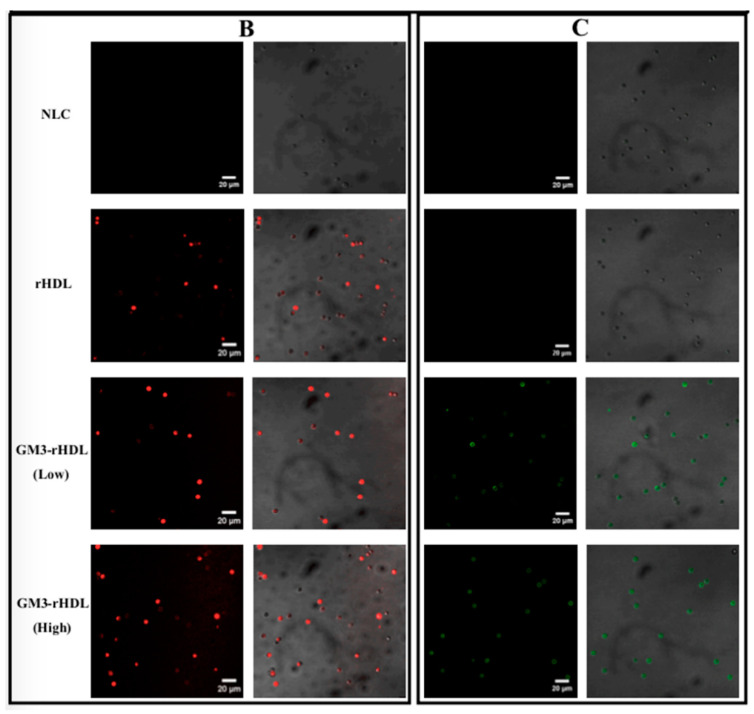
Validation of the presences of ApoA-I and GM3 in GM3-rHDL nanoparticles via a microsphere-based method. (**A**) Schematic diagram showing the microsphere-based method. The nanoparticles were reacted with anti-ApoA-I-biotin and subsequently conjugated with streptavidin-coated silica microspheres for the following validations via fluorescence staining and confocal microscopy. The validation of ApoA-I by Nile red staining is showed on the left and the validation of GM3 by staining with anti-GM3 IgM plus AlexaFlour488-conjugated anti-IgM 2nd antibody is showed on the right. (**B**) Representative confocal images of nanoparticles-bearing microspheres stained by Nile Red. (**C**) Representative confocal images of nanoparticles-bearing microspheres stained by AlexaFlour488. Panels from top to bottom: nanostructured lipid carrier (NLCs), rHDL, GM3_L_-rHDL, and GM3_H_-rHDL, respectively. Left panels: fluorescence images; right panels: the merged images from the fluorescence and DIC images.

When nanoparticles are prepared from a mixture of multiple molecule types by any method, a question about whether the major components are really included in the nanoparticles (e.g., the inclusion of ApoA-1 in rHDL nanoparticles and the inclusions of both ApoA-1 and GM3 in GM3-rHDL nanoparticles) automatically arises. To answer this question, we developed a simple, microsphere-based method (Figure 2A). The nanoparticles were specifically coupled onto the surfaces of the streptavidin-coated microspheres via biotinylated anti-ApoA-1 antibody (middle panel of Figure 2A); for detection of the presence of ApoA-1 in nanoparticles, the samples were then fluorescently stained in red with Nile Red for confocal microscopic imaging (left panel of Figure 2A); for detection of the presence of GM3 in nanoparticles, the samples were fluorescently (in green) labeled with AlexaFluor488-conjugated anti-IgM antibody specifically via anti-GM3 IgM for immunofluorescence imaging (right panel of Figure 2A). Therefore, only the presence of ApoA-1 (or GM3) in nanoparticles could cause the attachment of nanoparticles on microspheres making the microspheres red (or green) under confocal microscope. The confocal microscopic images (Figure 2B,C) show that the microspheres have no fluorescence in NLC group, only red fluorescence in rHDL group, and both red and green fluorescence in GM3_L_-rHDL and GM3_H_-rHDL groups. The data provides solid evidence confirming the inclusion of only ApoA-1 in rHDL nanoparticles and the inclusions of both ApoA-1 and GM3 in GM3-rHDL nanoparticles. 

### 2.3. Evaluation of In Vitro Anti-Atherosclerotic Efficacy of Gm3-Rhdl Nanoparticles via Cellular Experiments

**Figure 3:**
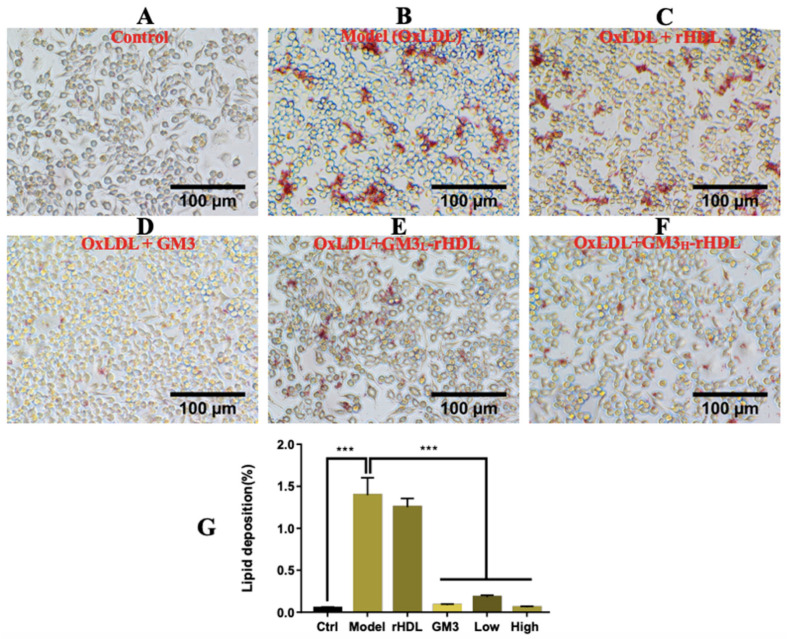
Inhibitory effects of GM3-rHDL nanoparticles on lipid deposition in macrophages. Deposited lipids in macrophages were stained with Oil Red O. (**A**) No stimulation/treatments. (**B**) OxLDL stimulation (100 μg/mL) for the induction of lipid deposition. (**C**) OxLDL stimulation and simultaneous rHDL treatment. (**D**) OxLDL stimulation and simultaneous treatment with exogenous GM3 (9 μg/mL). (**E**) OxLDL stimulation and simultaneous treatment with GM3_L_-rHDL nanoparticles at a GM3 concentration of 1.5 μg/mL. (**F**) OxLDL stimulation and simultaneous treatment with GM3_H_-rHDL nanoparticles at a GM3 concentration of 9 μg/mL. (**G**) Quantitative analysis (Average percentage of the lipid deposition area to the total cellular area in an image; *** *p* < 0.001 compared with the control or the model; *n* = 3).

Lipid deposition in macrophages which subsequently induces foam cell formation is a well-known key event in atherogenesis. Cultured macrophages treated with oxidized low-density lipoprotein (oxLDL) to induce intracellular lipid deposition which can be stained by Oil Red O are a widely used cellular model for the evaluation of in vitro anti-atherosclerotic efficacy of tested drugs. Prior to in vivo experiments, the in vitro anti-atherosclerotic efficacy of GM3-rHDL nanoparticles was evaluated via the atherosclerotic cellular model (Figure 3). Approximately 100 μg/mL of oxLDL induced significant lipid deposition in mouse RAW264.7 macrophages compared to the control (Figure 3A,B), confirming the successful establishment of the cellular model. The oxLDL-induced lipid deposition had no observable changes after rHDL treatment (Figure 3C), whereas each of GM3, GM3_L_-rHDL, and GM3_H_-rHDL obviously lowered/reversed the oxLDL-induced lipid deposition (Figure 3D–F, respectively); this was further confirmed by the quantitative analysis (Figure 3G). The data implies that both GM3 alone and GM3-rHDL nanoparticles can exert anti-atherosclerotic efficacy in vitro.

### 2.4. Evaluation of the Biocompatibility of GM3-rHDL Nanoparticles in Mouse Blood

**Figure 4:**
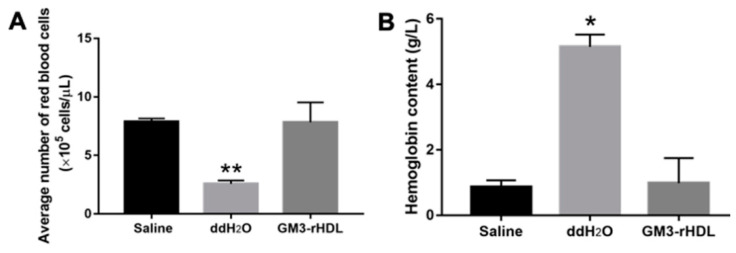
Biocompatibility of GM3-rHDL nanoparticles in ApoE^−/−^ mouse blood. (**A**) Effects of saline (0.9% NaCl), double distilled water (ddH_2_O), and GM3_H_-rHDL nanoparticles on the number of red blood cells in the pellet after 48 h of deposition. (**B**) Effects of saline, ddH_2_O, and GM3_H_-rHDL nanoparticles on the content of hemoglobin in suspension after 48 h of deposition. * *p* < 0.05, ** *p* < 0.01 (*n* = 3) compared with the control (the saline group).

Prior to the animal experiments, the biocompatibility of GM3-rHDL nanoparticles in blood was evaluated by testing the effect of GM3_H_-rHDL nanoparticles on the number of red blood cells (RBCs) and the content of hemoglobin released from RBCs (Figure 4). Compared with the control (the saline group), double distilled water (ddH_2_O) induced a significant decrease of average RBC number in pellet and a significant increase of hemoglobin content in supernatant whereas GM3_H_-rHDL nanoparticles caused no significant changes in both cell number and hemoglobin content after the 48-h incubation with mouse blood, implying the good biocompatibility of GM3-rHDL nanoparticles in blood.

### 2.5. Evaluation of In Vivo Anti-Atherosclerotic Efficacy of Gm3-Rhdl Nanoparticles via Animal Experiments

**Figure 5:**
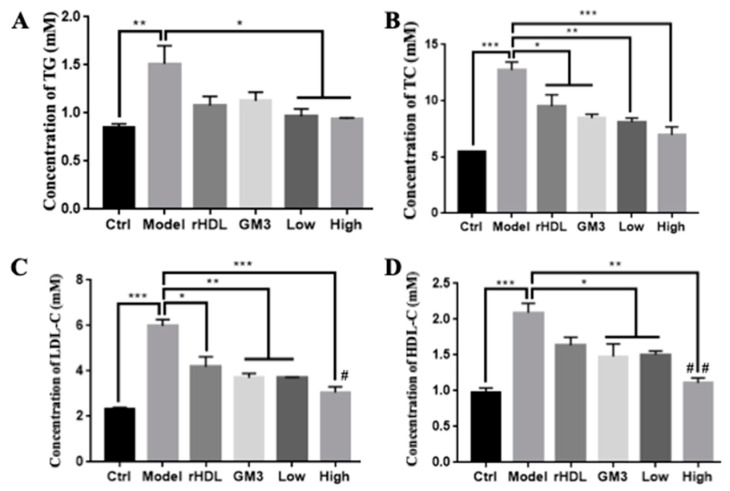
Inhibitory effects of GM3-rHDL nanoparticles on the high fat diet-induced increases in concentrations of main blood lipids in ApoE^−/−^ mice. The mice were fed a chow diet (ctrl) for eight weeks or a high-fat diet in combination with no treatments (model) or with a treatment (once every three days) of rHDL, ganglioside GM3 (1.2 mg/kg), GM3_L_-rHDL nanoparticles (0.2 mg/kg GM3), or GM3_H_-rHDL nanoparticles (1.2 mg/kg GM3), respectively for eight weeks. (**A**) Triglyceride (TG). (**B**) Total cholesterol (TC). (**C**) LDL-cholesterol (LDL-C). (**D**) HDL-cholesterol (HDL-C). * *p* < 0.05, ** *p* < 0.01, *** *p* < 0.001 (*n* = 6) compared with the control or with the model; ^#^ *p* < 0.05, ^##^ *p* < 0.01 (*n* = 6) compared with the GM3 group.

**Figure 6:**
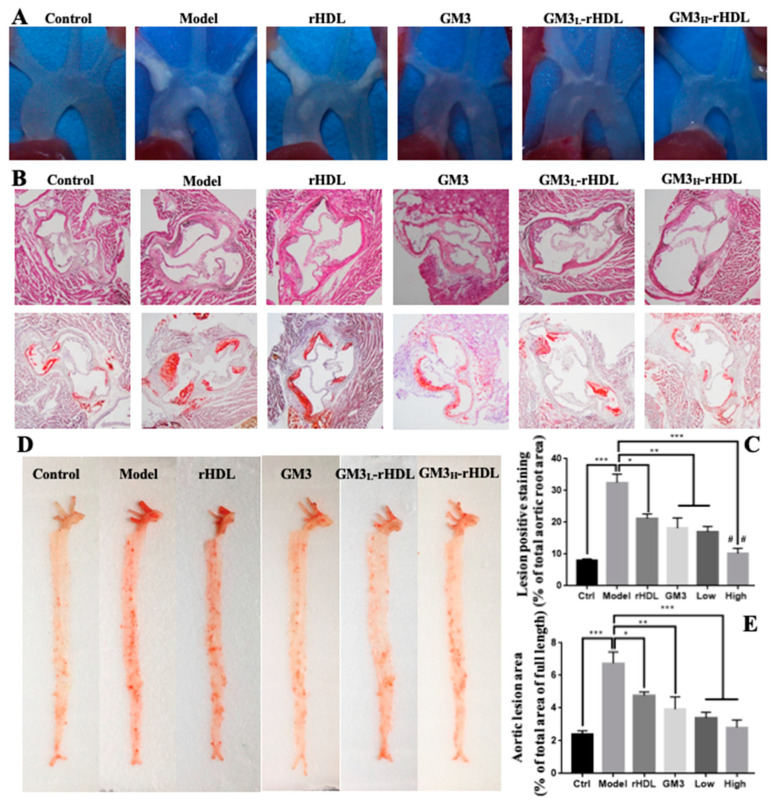
Inhibitory effects of GM3-rHDL nanoparticles on the formation of atherosclerotic lesions in ApoE^−/−^ mice fed a high-fat diet. The mice were fed a chow diet (ctrl) for eight weeks or a high-fat diet in combination with no treatments (model) or with a treatment (once every three days) of rHDL, ganglioside GM3 (1.2 mg/kg), GM3_L_-rHDL nanoparticles (0.2 mg/kg GM3), or GM3_H_-rHDL nanoparticles (1.2 mg/kg GM3), respectively for eight weeks. (**A**) Aortic arches (red arrows indicate atherosclerotic plaques). (**B**) Aortic root slices with H&E staining (upper panels) or Oil Red O staining (bottom panels). (**C**) Quantification of atherosclerotic lesions in aortic root slices with Oil Red staining. (**D**) Full-length aorta containing most of the aortic arch, thoracic aorta, and abdominal aorta with Oil Red O staining. (**E**) Quantification of atherosclerotic lesions in full length aorta with Oil Red O staining. * *p* < 0.05, ** *p* < 0.01, *** *p* < 0.001 (*n* = 6) compared with the control or with the model; ^##^ *p* < 0.01 (*n* = 6) compared with the GM3 group.

Then, ApoE-deficient (ApoE^−/−^) mouse fed a high-fat diet, a widely used animal model for atherosclerosis-related research [26], was recruited to evaluate the in vivo anti-atherosclerotic efficacy of GM3-rHDL nanoparticles. The mice were fed a high-fat diet in combination without treatment or with one of rHDL alone, GM3 alone, GM3_L_-rHDL, and GM3_H_-rHDL treatments for eight weeks. At the end, the concentrations of the main blood lipids in the mice including triglyceride (TG), total cholesterol (TC), LDL-cholesterol (LDL-C), and HDL-cholesterol (HDL-C) were measured (Figure 5), and the atherosclerotic plaques in aortic arches, aortic roots, and full-length aortas were imaged and quantified (Figure 6). Compared with the mice in the control group (i.e., the ApoE^−/−^ mice fed a chow diet for eight weeks and with no treatments), the mice in the model group (i.e., the ApoE^−/−^ mice fed a high-fat diet for eight weeks and with no treatments) displayed a dramatic increase in the level of main lipids (the second column in each graph of Figure 5) and in amount/area of atherosclerotic plaques (the second column/panel in each graph of Figure 6), confirming the successful establishment of the atherosclerotic animal model. Compared with the mice in the model group, the mice in the rHDL alone, GM3 alone, GM3_L_-rHDL, and GM3_H_-rHDL groups had gradually decreasing levels of main lipids (particularly the atherogenic lipids TC and LDL-C; columns 3–6 in each graph of Figure 5) and of atherosclerotic lesions (columns/panels 3–6 in each graph of Figure 6) although the difference between the rHDL group and the model group still is not statistically significant in TG and HDL-C (Figure 5A,D) and the difference between the GM3 group and the model group remained statistically nonsignificant in TG (Figure 5A). These data showed that all of rHDL, GM3, and GM3-rHDL nanoparticles could downregulate blood lipids and inhibit the formation of atherosclerotic lesions, implying the in vivo anti-atherosclerotic efficacy of these nanoparticles but to different extents. 

### 2.6. Validation of the Cell/Tissue-Targeting Ability of rHDL and GM3-rHDL Nanoparticles

**Figure 7:**
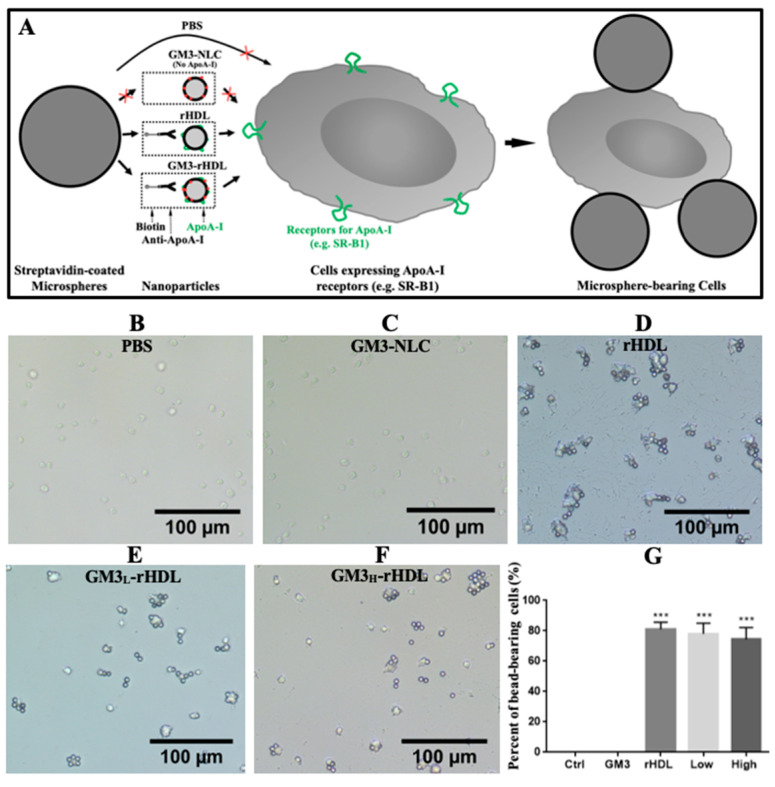
In vitro validation of cell-targeting ability of rHDL and GM3-rHDL nanoparticles via a microsphere-based method. (**A**) Schematic diagram showing the microsphere-based method. The nanoparticles were reacted with anti-ApoA-I-biotin, subsequently conjugated with streptavidin-coated silica microspheres, and incubated with RAW264.7 microphages. (**B**–**F**) Representative images of microsphere-bearing cells in different groups including PBS buffer, GM3-NLC, rHDL, GM3_L_-rHDL, and GM3_H_-rHDL, respectively. (**G**) Quantitative analysis (percent of microsphere-bearing cells in all macrophages). *** *p* < 0.001 (*n* = 6) compared with the control.

To test the cell-targeting ability of rHDL and GM3-rHDL nanoparticles, the streptavidin-coated microspheres which are observable under an optical microscope were utilized again. Via biotinylated anti-ApoA-1 antibody, only the ApoA-1-containing nanoparticles (i.e., rHDL and GM3-rHDL) could be coupled onto the streptavidin-coated microspheres, and only the microspheres with rHDL or GM3-rHDL nanoparticles could be recognized by and bound to the cells (e.g., macrophages) expressing receptors for ApoA-1 (e.g., scavenger receptor class B type 1 or SR-B1), based on which the cell-targeting ability of rHDL and GM3-rDHL nanoparticles could be determined by counting the microsphere-bearing cells (Figure 7A). Our data showed that no microspheres were coupled onto the surfaces of mouse RAW264.7 macrophages in the controls (i.e., the PBS group in Figure 7B and the group of GM3-NLC nanoparticles in Figure 7C) whereas many cells recognized at least one microsphere in the rHDL group (Figure 7D) and in the GM3-rHDL groups (Figure 7E,F). Quantitative analysis showed that the average percentage of microsphere-bearing cells was approximately 80% (Figure 7G). The cell experimental data provides evidence supporting the macrophage-targeting ability of rHDL and GM3-rHDL nanoparticles.

**Figure 8:**
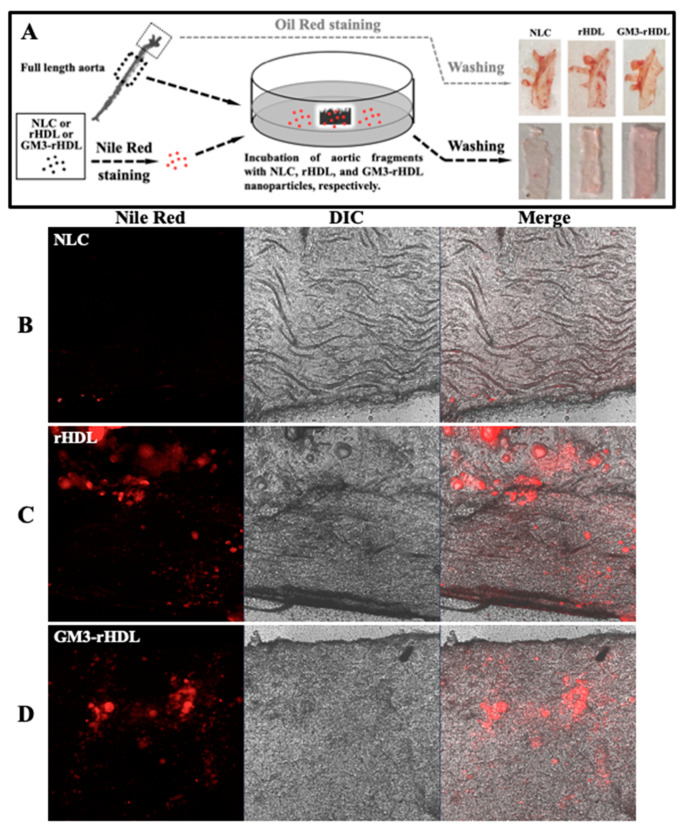
In vitro validation of atherosclerotic plaque-targeting ability of rHDL and GM3-rHDL nanoparticles. (**A**) Schematic diagram showing the experimental process. One fragment of an aorta is stained with Oil Red in order to confirm the formation of atherosclerotic plaques; a nearby fragment of the same aorta is incubated with an indicated nanoparticle pre-stained with Nile Red for determining the plaque-targeting ability of nanoparticles. The right panel shows the images of the entire fragments. (**B**–**D**) Representative confocal microscopic images of local areas of the aortic fragments incubated with NLC (**B**), rHDL (**C**), and GM3-rHDL (**D**), respectively, which were pre-stained with a fluorescent dye for lipids (Nile Red). The aortas were from the ApoE^−/−^ mice fed a high fat diet. Panels from left to right: fluorescent, DIC, and merged images, respectively.

To test the tissue-targeting ability of rHDL and GM3-rHDL nanoparticles, aortic fragments from the mice fed a high fat diet were utilized. In this experiment, one fragment was stained with Oil Red to confirm the formation of atherosclerotic plaques and the other fragment of the same aorta was incubated with the nanoparticles pre-stained with Nile Red to determine the atherosclerotic plaque targeting ability of the nanoparticles (Figure 8A). Red plaques could be clearly observed on all Oil Red-stained fragments (the upper row of right panel in Figure 8A). Compared with the control (the fragment incubated with Nile Red-treated NLC nanoparticles), the fragments incubated with Nile Red-stained rHDL/GM3-rHDL nanoparticles display a light red color (the lower row of right panel in Figure 8A). To take a microscopic observation, local areas of the fragments incubated with different nanoparticles were imaged by confocal microscopy. We found that red fluorescence was observed on the fragments incubated with Nile Red-stained rHDL (Figure 8B) or GM3-rHDL (Figure 8C) nanoparticles but not on the fragment incubated with Nile Red-treated NLC nanoparticles (Figure 8D). The data implies that both rHDL and GM3-rHDL nanoparticles have the atherosclerotic plaque-targeting ability.

### 2.7. Verification of the pH-Responsive Release of GM3 from GM3-rHDL Nanoparticles

**Figure 9:**
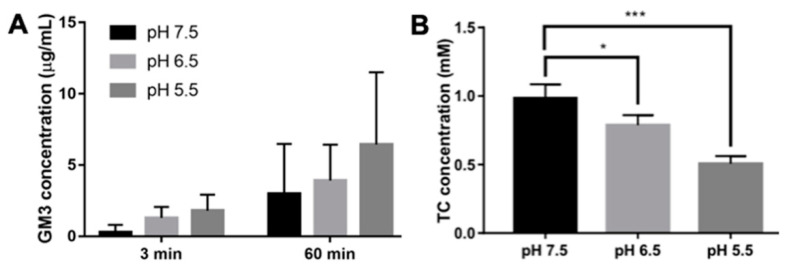
Solution acidification enhances the release of GM3 and cholesterol in GM3-rHDL particles. (**A**) The concentration of the released GM3 from GM3-rHDL particles at different pH values for 0 and 1 h (*n* = 3). (**B**) The concentration of total cholesterol (TC) remaining in GM3-rHDL particles at different pH values for 1 h. * *p* < 0.05 and *** *p* < 0.001 (*n* = 4).

To test the pH-sensitive release of free GM3 from GM3-rHDL nanoparticles, GM3-rHDL nanoparticles were put into the solutions at different pH values, and the concentration of GM3 molecules released from nanoparticles into the solution was measured by HPLC. The result shows that solution acidification caused a higher concentration of released GM3 compared with the neutral pH7.5 solution (Figure 9A), implying the pH-sensitive release of GM3. After treating with the solutions at various pH values, the concentrations of total cholesterol in GM3-rHDL nanoparticle remnants also were quantified. The data shows that solution acidification also induced the statistically significant loss of total cholesterol in GM3-rHDL nanoparticles (Figure 9B).

## 3. Discussion

This study had the following major aims: (a) to further confirm the anti-atherosclerotic efficacy of exogenous GM3 which has been reported in our previous study [13]; (b) to test the probability of using a lower dose of exogenous GM3 to reach a similar or even better anti-atherosclerotic efficacy via a drug nanocarrier (here, rHDL was recruited as a representative due to its atherosclerotic plaque-targeting ability [27]); and (c) to test the specific cell/tissue-targeting ability of the GM3-rHDL nanoparticles. Nanometer-sized rHDL and GM3-rHDL particles (~150–230 nm) were successfully prepared, characterized (Table 1 and Figure 1 and Figure 2), and utilized to realize the abovementioned aims. 

Cellular experiments show that compared with the model group free GM3 molecules induced a significant decrease of lipid deposition in oxLDL-treated macrophages (Figure 3D and the forth group in Figure 3G), implying the in vitro anti-atherosclerotic efficacy of free GM3 molecules. Animal experiments show that compared with the model group, free GM3 molecules (1.2 mg/kg/3d) also induced a significant decrease in blood lipids (particularly cholesterol; the fourth group on each graph in Figure 5) and atherosclerotic plaque areas at different aortic locations (aortic arch, aortic root, and aortic full length, respectively; the fourth group in Figure 6A–E) in ApoE^−/−^ mice fed a high-fat diet, implying the in vivo anti-atherosclerotic efficacy of free GM3 molecules. These data further confirm our previous study in which more than 1 mg/kg/3d of exogenous GM3 (i.e., the middle-dose and high-dose groups) could induce a statistically significant anti-atherosclerotic efficacy [13]. In both studies, the exogenous GM3 treatments were offered during the high fat diet to evaluate its effect on atherosclerotic development. Further studies about the effect of exogenous GM3 on advanced atherosclerotic plaques by offering GM3 after a high fat diet will be needed. Interestingly, in both studies, we also found that exogenous GM3 alone was able to induce the lowering of blood lipids in mice fed a high fat diet. It implies that exogenous GM3 probably can influence the lipid metabolism e.g., in the liver because the inhibitory effect of exogenous GM3 on the secretion of apoB-100 (the major structural apolipoprotein in very low-density lipoprotein or VLDL) in liver cells has been reported previously [28].

Similarly, compared with the model group, both GM3_L_-rHDL and GM3_H_-rHDL nanoparticles caused a significant decrease of lipid deposition in macrophages (Figure 3E,F and the last two groups in Figure 3G) and a significant decrease in blood lipids (the last two groups on each graph in Figure 5) and atherosclerotic plaque areas at different locations of aortas (the last two groups in Figure 6A–E) in ApoE^−/−^ mice fed a high fat diet, implying the in vitro and in vivo anti-atherosclerotic efficacy of GM3-rHDL nanoparticles. Compared with the free GM3 group, GM3_H_-rHDL nanoparticles exerted an enhanced anti-atherosclerotic effect although both of them used the same GM3 dose (1.2 mg/kg/3d) for animal experiments. Moreover, GM3_L_-rHDL nanoparticles at a sixfold lower GM3 dose (0.2 mg/kg/3d) also had a similar or even better efficacy than free GM3 molecules (1.2 mg/kg/3d) whereas 0.4 mg/kg/3d of free GM3 molecules (i.e., the low dose group) could not cause a statistically significant anti-atherosclerotic effect in our previous study [13]. These data prove that the dose of exogenous GM3 can drop but still maintain a similar or even better anti-atherosclerotic efficacy by using rHDL as a delivery system (i.e., GM3-rHDL nanoparticles).

Gangliosides including GM3 are natural molecules expressed in almost all animal cells/tissues. It is challenging to track exogenous GM3 without specific labeling (e.g., isotope/fluorophore labeling) in animals. Therefore, the animal experiments for evaluating the dynamic changes of exogenous GM3 in blood (pharmacokinetic parameters) and the distribution of exogenous GM3 in various tissues after a single drug administration were not performed in the present study, among which the latter (i.e., the tissue distribution data) is generally used to provide useful information for determining the tissue-targeting ability of drugs. To answer the question about the drug targetability, we particularly designed two in vitro experiments to determine the cell- and tissue-targeting abilities of the nanoparticles without isotope/fluorophore labeling by investigating the interactions of microsphere-coupled nanoparticles and free nanoparticles with macrophages (Figure 7A) and aortic fragments (Figure 8A), respectively. The data show that only the ApoA-I-bearing nanoparticles (i.e., rHDL and GM3-rHDL) had both macrophage-targeting (Figure 7B–G) and atherosclerotic plaque-targeting (Figure 8B–D) abilities. The data imply that GM3-rHDL nanoparticles can specifically target macrophages in atherosclerotic plaques via ApoA-I because the cells in atherosclerotic plaques (e.g., macrophages) generally express a relatively high level of ApoA-I receptors (e.g., scavenger receptor class B type 1 or SR-B1). It has been reported that SR-B1 can bind natural plasma HDL with high affinity [29,30,31,32], and serves as the optimal target of rHDL nanoparticles [33,34,35]. On the other hand, the rHDL-mediated cell/tissue targetability also explains why GM3-rHDL nanoparticles at a 6-fold lower GM3 dose could reach a similar or even better anti-atherosclerotic efficacy than free GM3 molecules at a high dose.

It is well known that atherosclerotic lesions generally cause a local acidic extracellular microenvironment [36,37]. Once GM3-rHDL nanoparticles enter atherosclerotic plaques, these nanoparticles will be in an acidic solution. Recently, we found that solution acidification can alter the biomechanical property of low-density lipoproteins (LDL) by changing the secondary structures of LDL proteins and depleting a part of LDL lipids [38]. Therefore, we speculated that an acidic solution may also remove lipids including GM3 from the GM3-delivering rHDL nanoparticles (i.e., improve the release of GM3 from GM3-rHDL nanoparticles). To test this speculation as the forth aim of this study, GM3-rHDL nanoparticles were suspended in solutions at different pH values, and then the concentrations of GM3 released from the nanoparticles to the solutions (Figure 9A) and the concentrations of total cholesterol kept in the nanoparticle remnants (Figure 9B) were measured. The data show that solution acidification caused the pH-dependent increase in both GM3 and cholesterol released from the GM3-rHDL nanoparticles, implying that the pH-responsive release of free GM3 molecules is probably due to the acidification-induced removal of other major lipids (e.g., cholesterol, phospholipids, etc.) of rHDL. Currently, it is unclear how solution acidification triggers the abjunction of some lipids from plasma lipoproteins (e.g., LDL, HDL/rHDL, etc.).

**Figure 10:**
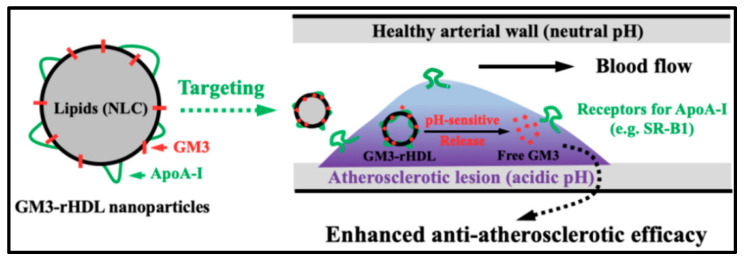
Schematic diagram showing the lesion targeting of GM3-rHDL nanoparticles and pH-responsive release of free GM3 under an acidic condition in atherosclerotic plaques.

Taken together, the GM3-rHDL nanoparticle using ApoA-I-containing rHDL nanoparticle as a nanocarrier could exert an enhanced anti-atherosclerotic efficacy due to its macrophage/lesion-targeting ability via the specific interaction between ApoA-I and cellular receptors (e.g., SR-B1) as well as the pH-responsive release of free GM3 after GM3-rHDL nanoparticles get into the acidic solution in atherosclerotic plaques from the neutral solution in blood circulation or in healthy artery intima (Figure 10). Therefore, the GM3 dose for producing a statistically significant anti-atherosclerotic efficacy can be dramatically lowered. Moreover, the potential side effects of exogenous GM3 may be avoided due to the dramatic decrease of the GM3 dose, which is outside the purview of the present study and needs to be confirmed in the future. The development of the GM3-rHDL nanoparticle may pave the way for the application of exogenous GM3 as a clinical drug. On the other hand, the GM3-rHDL nanoparticle has the possibility of being recruited as a drug delivery system with atherosclerotic targetability and enhanced anti-atherosclerotic efficacy. Considering the potential anti-tumor efficacy of exogenous GM3 and the tumor-targeting ability of rHDL, the GM3-rHDL nanoparticle also has the possibility of being recruited as a drug delivery system with tumor targetability and enhanced anti-tumor efficacy.

## 4. Materials and Methods

### 4.1. Reagents, Cells, and Cell Culture

Egg phospholipid (PC, Lipoid E80) for nanostructured lipid carriers (NLC) reconstitution were purchased from Lipoid GmbH (Ludwigshafen, Germany). Cholesterol for NLC reconstitution and streptavidin were from Solarbio Science & Technology Co. (Shanghai, China). Octadecylamine, cholesteryl oleate, and glycerol trioleate for NLC reconstitution, as well as Oil Red O and Nile Red for lipid staining, were purchased from Sigma (St. Louis, MO, USA). Human ApoA-I for reconstituted high-density lipoprotein (rHDL) synthesis and biotin-conjugated anti-ApoA-I antibody (anti-ApoA-I-biotin) for specifically recognizing ApoA-I were purchased from Cloud-clone (Katy, TX, USA). Ganglioside GM3 was from AdipoGen Life Sciences (Liestal, Switzerland). Human ox-LDL for foam cell induction of macrophages was from Yiyuan Biotechnologies (Guangzhou, China). Carboxyl silica microspheres were from Bangs Laboratories, Inc. (Fishers, IN, USA). Anti-GM3 IgM and AlexaFluor488-conjugated goat anti-mouse IgM antibodies were from Amsbio (Milton Park Abingdon, UK) and Invitrogen (Carlsbad, CA, USA), respectively. Other reagents were all of analytical/chromatographic grade.

Mouse RAW264.7 macrophages, purchased from the Cell Bank of the Chinese Academy of Sciences (Shanghai, China), were routinely cultured in RPMI 1640 Media (Gibco) and supplemented with 10% FBS, 100 Unit/mL penicillin and 100 μg/mL streptomycin. The cells at passage ~5 were used in the experiments.

### 4.2. Preparations of rHDL and GM3-rHDL

The thin film dispersion method was used to prepare rHDL and GM3-rHDL as previously described with minor revision [25]. Briefly, 22.5 mg PC, 10 mg cholesteryl oleate, 7.5 mg glycerol trioleate, 5 mg cholesterol, and 2.5 mg octadecylamine were dissolved in 7.5 mL of methanol/chloroform (1:1, *v*/*v*) to get a lipid mixture. The lipid mixture was then mixed with 0, 0.375, and 2.250 mg GM3 in methanol/chloroform (1:2, *v*/*v*), respectively and dried under vacuum at 45 °C for 1 h to form a dried thin film in an egg-plant flask. The organic solvent was removed by putting the flask under vacuum overnight. After adding 15 mL of 0.02 M Tris buffer (pH 8.0) containing 10 mg sodium cholate and drying again at 45 °C for 1 h, the film was dispersed via vortexing for 15 min and ultrasonicated in an ice bath. Next, the suspension was filtered through a 0.22 μm sterile filter to obtain nanostructured lipid carriers (NLC) or GM3-NLC at a low or high GM3 concentration. Then, 50 µg ApoA-I was added into 2 mL of NLC or GM3-NLC and incubated at 37 °C for 48 h by shaking at 150 rpm. After dialyzing in a 10 kDa dialysis bag (Solarbio Science & Technology Co.) at 4 °C for two days to remove free GM3 and sodium cholate, the rHDL without GM3, GM3-rHDL at a low GM3 concentration (GM3_L_-rHDL), and GM3-rHDL at a high GM3 concentration (GM3_H_-rHDL) were prepared and stored at 4 °C or subjected to the downstream experiments.

### 4.3. Imaging and Size Measurement of Rhdl and Gm3-Rhdl Nanoparticles

A dynamic light scattering (DLS) Analyzer (Zetasizer nano zs90, Malvern, UK) was used to measure the mean size and polydispersity index (PDI) of rHDL, GM3_L_-rHDL and the GM3_H_-rHDL nanoparticles as previously reported [25]. These nanoparticles pre-stained with 2% (*w*/*v*) uranyl acetate were imaged by a transmission electron microscope (JEOL JEM-2100 TEM, Japan).

### 4.4. Determination of Entrapment Efficiency (Ee) and Drug Loading Efficiency (Dl) of Gm3-Rhdl

The contents of GM3 loaded in GM3_L_-rHDL and GM3_H_-rHDL were measured by the HPLC-UV method using a Waters e2695 Alliance series (Waters Corp., Milford, MA, USA). A symmetry C18 column (250 × 4.6 mm, 5 μm) was used at 35 °C. A mixture of 80% methanol (*v*/*v*) and 20% acetonitrile (*v*/*v*) of chromatographic grade was utilized as the mobile phase (flow rate: 1 mL/min). The detected wavelength was 260 nm. The calculations of EE and DL were based on the following equations [25,39]: EE (%) = W/W_t_ × 100% and DL (%) = Q/Q_t_ × 100%, where W and Q are the amount of GM3 in each drug carrier whereas W_t_ and Q_t_ are the total amount of the feeding GM3 and the feeding materials.

### 4.5. Preparation of Streptavidin-Coated Silica Microspheres

Streptavidin-coated silica microspheres/beads were prepared as reported previously [40,41]. Briefly, approximately 1 mg carboxyl silica microspheres in a diameter of ~5 μm were washed twice with 1 mL of 15 mM 2-(*N*-morpholino) ethanesulfonic acid (MES buffer; pH 6.0) and suspended in 100 μL of 15 mM MES buffer (pH 6.0). The suspended microspheres were mixed with 100 μL of 10 mg/mL 1-ethyl-3-(3-dimethylaminopropyl)carbodiimide hydrochloride (EDC; pH 6.0 in 15 mM MES buffer) and reacted at room temperature for 30 min. After washing with 1 mL of 15 mM MES buffer (pH 6.0), the microsphere pellets were incubated with streptavidin in MES buffer (at a final concentration of 50 μg/mL) at room temperature overnight. After washing with MES buffer to remove potential free streptavidin molecules, the streptavidin-coated silica microspheres were prepared and stored for the following experiments.

### 4.6. Confirmation of the Presences of Gm3 and Apoa-I in Gm3-Rhdl Nanoparticles by Using A Microsphere-Based Method

A method based on micrometer-sized beads (or microspheres) was used to determine the presence of GM3 and ApoA-I in GM3-rHDL nanoparticles. The prepared streptavidin-coated silica microspheres/beads were diluted to 1 × 10^6^ beads/mL in biotin-streptavidin binding buffer, washed with PBS, and suspended in 100 μL PBS. For the confirmation of ApoA-I presence in GM3-rHDL, approximately 1 mL of each of four samples (NLC as a control, rHDL, GM3_L_-rHDL, and GM3_H_-rHDL, respectively) was incubated with 5 μL of anti-ApoA-I-biotin (200 μg/mL) at room temperature by shaking at 80 rpm for 1 h and subsequently with 100 μL streptavidin-coated silica microspheres in PBS at 37 °C for 12 h. After washing, the microspheres were incubated with Nile Red (10 μg/mL) in DMSO at 37 °C for 30 min. After washing with PBS, the microspheres in petri dishes were imaged by a LSM710 confocal microscope (Carl Zeiss, Oberkochen, Germany). 

For the confirmation of GM3 presence in GM3-rHDL, approximately 1 mL of each of the samples was incubated with 5 μL of anti-ApoA-I-biotin (200 μg/mL) at room temperature by shaking at 80 rpm for 1 h and subsequently with 100 μL streptavidin-coated silica microspheres in PBS at 37 °C for 12 h. After washing with PBS, the beads were incubated successively with 1 μL anti-GM3 IgM (1:1000) at 37 °C for 1 h and with 1 μL AlexaFlour488-conjugated goat anti-mouse IgM antibody (1:500) at 37 °C for 1 h (PBS washing was also performed). After washing three times with PBS, the microspheres in petri dishes were imaged by confocal microscopy.

### 4.7. In Vitro Reversal of Ox-ldl-Induced Lipid Deposition in Macrophages by Gm3-rhdl Nanoparticles

Six groups of stimulation/treatment were categorized: (a) no stimulation/treatments (blank control); (b) ox-LDL stimulation (100 μg/mL) for the induction of lipid deposition; (c) ox-LDL stimulation + rHDL treatment; (d) ox-LDL + GM3 (9 μg/mL); (e) ox-LDL + GM3_L_-rHDL (a GM3 concentration of 1.5 μg/mL); and (f) ox-LDL + GM3_H_-rHDL (a GM3 concentration of 9 μg/mL). After the stimulation/treatment in a 5% CO_2_ incubator at 37 °C for 24 h and rinsing twice with PBS, mouse RAW264.7 macrophages were fixed with 2.5% glutaraldehyde at room temperature for 10 min, rinsed again, and treated with 60% isopropanol for 1−2 min. After isopropanol removal, the cells were stained with Oil Red O at room temperature for 20 min and treated with 60% isopropanol for 30 s twice. After rinsing twice with PBS, the cells were imaged by an inverted microscope (Nikon LH-M100CB, Tokyo, Japan). ImageJ software was used to quantify the total area (in red) of lipid deposition in cells and the total area of cells for quantitative analysis.

### 4.8. Evaluation of the Biocompatibility of Gm3-Rhdl Nanoparticles in Mouse Blood

Blood samples were from ApoE^−/−^ C57BL/6 mice. Three groups were categorized and treated as follows: (a) the saline group: 100 μL of blood + 320 μL of 0.9% NaCl; (b) the ddH_2_O group: 100 μL of blood + 200 μL of double distilled water (ddH_2_O) + 120 μL of 0.9% NaCl; (c) the GM3-rHDL group: 100 μL of blood + 300 μL of 0.9% NaCl + 20 μL of GM3_H_-rHDL. The cells in the mixtures were allowed to deposit for 48 h at room temperature. Subsequently, 10 μL of pellet was taken from each group and diluted in 1 mL of 0.9% NaCl for the measurement of the number of red blood cells (RBCs) via a blood cell counting plate; at the same time, 50 μL of supernatant was taken for the measurement of hemoglobin content by using the Hb Kit (Nanjing Jiancheng Bioengineering Institute Ltd., Nanjing, China) and a UV-5100 spectrophotometer (Metash Instruments, Shanghai, China) at 540 nm.

### 4.9. Animals, Diet, and Treatments

Eight-week-old male ApoE^−/−^ C57BL/6 mice, purchased from Beijing Vital River Laboratory Animal Technology Co., Ltd. (Beijing, China), were recruited. To establish the atherosclerotic mouse model, the mice were fed a high-fat diet (Hunan SJA Lab Animal Ltd., Changsha, China) which contained 21% fat, 0.15% cholesterol, and basic forage. Ethics approval for this study was obtained from the Ethics Committee of Jiangxi University of Chinese Medicine (approval number: JZLLSC2017-205; 28 December 2017). All animal experiments were performed in full compliance with the National Institute of Health Guide for the Care and Use of Laboratory Animals.

For the in vivo experiment, 36 ApoE^−/−^ mice were randomly divided into six groups (*n* = 6 in each group) including a control group (mice fed a chow diet for eight weeks), a model group (mice fed a high-fat diet for eight weeks), and the groups fed a high-fat diet for eight8 weeks and meanwhile intravenously treated once every three days with ~100 μL of rHDL, GM3 (1.2 mg/kg), GM3_L_-rHDL containing 0.2 mg/kg GM3 and GM3_H_-rHDL containing 1.2 mg/kg GM3, respectively, via tail vein injection.

### 4.10. Measurements of Major Blood Lipids

After 8 h of fasting at the end of the eight-week treatments, the whole blood of each mouse was collected in an EDTA-containing EP tube and centrifugated at 3000 rpm for 10 min to obtain the serum. An automatic biochemical analyzer (Beckman Coulter AU480, Brea, CA, USA) was utilized to measure the blood concentrations of the major lipids, including total cholesterol (TC), triglyceride (TG), low density lipoprotein-cholesterol (LDL-C), and high density lipoprotein-cholesterol (HDL-C) by using different Kits (all from Anhui Iprocom Biotechnology Co., Ltd.; Anhui, China).

### 4.11. Imaging and Quantification of Atherosclerotic Lesions in Aortic Arch, Aortic Root, and Full-Length Aorta

The imaging and quantification of atherosclerotic lesions in different parts of aorta were performed as reported previously [13]. For in situ imaging of the atherosclerotic lesions in aortic arch of each mouse, after blood collection and perfusion, a blue background was put under the heart and aortic arch and a picture was taken immediately. Then, the heart coupling with entire aorta was taken from each mouse. For imaging of the atherosclerotic lesions in full length aorta, the part containing most of the aortic arch, thoracic aorta, and abdominal aorta were cut, fixed with 4% paraformaldehyde, opened longitudinally, stained with Oil Red O, washed with 60% propylene glycol, unrolled, and photographed. For imaging of the atherosclerotic lesions in aortic root, the heart containing aortic root was separated, frozen rapidly, embedded in tissue OCT-freeze medium, sliced (a thickness of ~7 μm), fixed with 95% ethanol, stained with hematoxylin-eosin or hematoxylin-Oil Red, and photographed. ImageJ software was used to process the images for quantitative analyses.

### 4.12. In Vitro Validation of the Macrophage Targeting of Rhdl and Gm3-Rhdl Nanoparticles by Using another Microsphere-Based Method

The prepared streptavidin-coated silica microspheres/beads were diluted to 1 × 10^6^ beads/mL in biotin-streptavidin binding buffer, washed with PBS, and suspended in 100 μL PBS. approximately 200 μL of each of five samples (PBS buffer as a control, rHDL, GM3-NLC, GM3_L_-rHDL, and GM3_H_-rHDL, respectively) was incubated with 1 μL of anti-ApoA-I-biotin (200 μg/mL) at room temperature for 1 h and subsequently with 200 μL streptavidin-coated silica microspheres in PBS at 37 °C by shaking at 80 rpm for 12 h. After washing three times with PBS, the microspheres were suspended in RPMI 1640 medium. Subsequently, mouse RAW264.7 macrophages in a petri dish were incubated at 37 °C for 1 h with the microspheres which were coated with anti-ApoA-I antibody and interacted with the five samples. After rinsing twice with PBS to remove free microspheres, the cells were fixed with 2.5% glutaraldehyde for 10 min. After rinsing again, the cells were subjected to confocal microscopy. For quantitative analysis, the percentage of microsphere-bearing macrophages were calculated.

### 4.13. In Vitro Validation of the Atherosclerotic Plaque Targeting of Rhdl and Gm3-Rhdl Nanoparticles

NLC, rHDL, and GM3-rHDL nanoparticles were fluorescently stained with Nile Red (a final concentration of 10 μg/mL) at 37 °C for 20 min by shaking at 80 rpm, washed at least three times with PBS by centrifugation at 12,000 rpm for 5 min, and suspended in a PBS buffer. After preparation of the full-length aortas from ApoE^−/−^ mice fed a high-fat diet, aortic fragments were cut from the thoracic aorta sections, and the aortic arch sections from the same full-length aortas were stained by Oil Red to confirm the formation of atherosclerotic plaques. The thoracic aortic fragments were opened longitudinally, immobilized on the substrate, and incubated with 1 mL PBS buffer (as a blank control, the data not shown) or the PBS buffer containing NLC (as a negative control), rHDL, and GM3-rHDL nanoparticles, respectively at 37 °C overnight by shaking at 80 rpm. After washing three times with PBS buffer, the aortic fragments were imaged by confocal microscopy.

### 4.14. Quantification of the Released Gm3 and ghe Remaining Total Cholesterol at Different Ph Values

For quantification of the released GM3, approximately 100 μL GM3-rHDL nanoparticles (30 μg GM3/100 μL) was mixed with 900 μL Tris-HCl buffer (pH 8.0) and the pH value was adjusted to pH 7.5, pH 6.5, and pH 5.5, respectively by using hydrochloric acid (HCl) diluent. After 3 min or 60 min of incubation, the solutions were filtered by the Amicon Ultra-0.5 mL, 100 kDa Centrifugal Filter Unit (Merck Millipore) at 14,000 rpm for 20 min according to the user’s manual. Then the filtrates were analyzed by HPLC for the measurement of released GM3 by using Agilent 1290 (Agilent Technologies, Santa. Clara, CA, USA). A ZORBAX ECLIPSE XDB-C18 column (150 × 4.6 mm, 5 μm) was used at 25 °C. Pure methanol of chromatographic grade was utilized as the mobile phase (flow rate: 1 mL/min). The detected wavelength was 260 nm.

For quantification of the remaining total cholesterol, approximately 100 μL GM3-rHDL nanoparticles (30 μg GM3/100 μL) was mixed with 900 μL Tris-HCl buffer (pH 8.0) and the pH value was adjusted to pH 7.5, pH 6.5, and pH 5.5, respectively by using hydrochloric acid (HCl) diluent. After 60 min of incubation, the solutions were centrifugated at 12,000 rpm and the pellets were subjected to the measurement of total cholesterol by using the LDL-C Kit (Nanjing Jiancheng Bioengineering Institute Ltd., Nanjing, China) and a UV-5100 spectrophotometer (Metash Instruments, Shanghai, China) at 546 nm.

### 4.15. Statistical Analysis

All data from at least three independent experiments are expressed as the mean ± SD. Statistical analyses were performed using paired Student’s *t*-test between two groups or one-way ANOVA among multiple groups. *p* < 0.05 was considered a statistically significant difference.

## Figures and Tables

**Table 1:** Mean size, PDI, Zeta potential, EE, and DL (mean ± SD, *n* = 3).

	rHDL	GM3_L_-rHDL	GM3_H_-rHDL
Mean size (nm)	161.3 ± 8.8	176.8 ± 9.2	209.6 ± 22.1
polydispersity index (PDI)	0.28 ± 0.02	0.22 ± 0.01	0.21 ± 0.05
Zeta potential (mV)	−14.58 ± 0.80	−22.22 ± 4.61	−13.82 ± 1.48
EE (%)	---	92.63 ± 3.57	80.16 ± 5.14
DL (%)	---	0.68 ± 0.03	3.39 ± 0.22

Abbreviations: PDI, polydispersity index; EE, entrapment efficiency; DL, drug loading efficiency; rHDL, reconstituted high-density lipoprotein.

## Data Availability

All data generated or analyzed during this study are included in this article.
